# Shaping Adoption and Sustained Use Across the Maternal Journey: Qualitative Study on Perceived Usability and Credibility in Digital Health Tools

**DOI:** 10.2196/59269

**Published:** 2024-10-01

**Authors:** Wei Ying Ng, Ni Yin Lau, V Vien Lee, Smrithi Vijayakumar, Qiao Ying Leong, Shu Qin Delicia Ooi, Lin Lin Su, Yung Seng Lee, Shiao-Yng Chan, Agata Blasiak, Dean Ho

**Affiliations:** 1 The N.1 Institute for Health National University of Singapore Singapore Singapore; 2 The Institute for Digital Medicine (WisDM) Yong Loo Lin School of Medicine National University of Singapore Singapore Singapore; 3 Department of Paediatrics Yong Loo Lin School of Medicine National University of Singapore Singapore Singapore; 4 Department of Obstetrics & Gynaecology Yong Loo Lin School of Medicine National University of Singapore Singapore Singapore; 5 Department of Obstetrics & Gynaecology National University Hospital Singapore Singapore; 6 Division of Paediatric Endocrinology Khoo Teck Puat-National University Children's Medical Institute National University Hospital Singapore Singapore; 7 Singapore Institute for Clinical Sciences Agency for Science, Technology and Research (A*STAR) Singapore Singapore; 8 Department of Biomedical Engineering National University of Singapore Singapore Singapore; 9 Department of Pharmacology Yong Loo Lin School of Medicine National University of Singapore Singapore Singapore; 10 The Bia-Echo Asia Centre for Reproductive Longevity and Equality Yong Loo Lin School of Medicine National University of Singapore Singapore Singapore

**Keywords:** maternal and child health, conception, pregnancy, perinatal care, postpartum, maternal care, obstetric care, user engagement, Unified Theory of Acceptance and Use of Technology, femtech

## Abstract

**Background:**

Maternal and child health outcomes are positively influenced by early intervention, and digital health (DH) tools provide the potential for a low-cost and scalable solution such as informational platforms or digital tracking tools. Despite the wide availability of DH tools out there for women from before to after pregnancy, user engagement remains low.

**Objective:**

This study aims to explore the factors that shape women’s DH adoption and sustained use across the maternal journey from preconception to postbirth, to improve user engagement with DH tools.

**Methods:**

One-hour semistructured qualitative interviews were conducted with 44 women from before to after pregnancy (age range 21-40 years) about their experiences with DH. This study is part of a larger study on women’s maternal experiences with health care and DH and focuses on the factors that affected women’s DH adoption and sustained use. Interviews were audio recorded, transcribed verbatim, and analyzed using inductive thematic analysis.

**Results:**

Five main themes and 10 subthemes were identified that affected women’s adoption and sustained use of DH tools. These included themes on their preexisting attitudes to DH, perceived ease of use, perceived usefulness, perceived credibility, and perceived value of the tool.

**Conclusions:**

The themes that emerged were fully or partially mapped according to the Unified Theory of Acceptance and Use of Technology 2 model. The applicability of the model and the need to consider specific cultural nuances in the Asian context (such as the importance of trust and social influence) are discussed. The interaction of the 5 themes with DH adoption and sustained use are explored with different themes being relevant at various points of the DH adoption journey. The insights gained serve to inform future DH design and implementation of tools for women to optimize their DH engagement and the benefits they derive from it.

**Trial Registration:**

ClinicalTrials.gov NCT05099900; https://clinicaltrials.gov/study/NCT05099900

## Introduction

The lifelong health of an individual can be linked to periods even before conception and birth, as maternal health and behaviors have been shown to influence child health [1]. Many countries including Singapore, have worked on improving the quality of maternal health care [2]. In recent years, the challenges in maternal health have shifted from lowering maternal and infant mortality rates to managing complicated health outcomes such as obesity in women before, during, and after pregnancy [2]. Unhealthy weight in women can have implications for both mothers (eg, attempts at conception, pregnancy-related complications, and mental health disorders) and their children (eg, increased risks of childhood obesity and developmental problems later in life) [1,2]. These are known as the “twin metabolic and mental health challenges” as characterized in the S-PRESTO and GUSTO mother-offspring cohort studies in Singapore [3,4]. Evidently, managing maternal health outcomes has both direct and indirect consequences on maternal and child health [1-4]. As such, early intervention in maternal health decisions, as early as preconception, has the potential to positively influence maternal and child health outcomes [2].

A multitude of digital health (DH) tools have been developed to improve multiple maternal and child outcomes [5-14]. DH tools can take the form of digital platforms, such as smartphones, websites, forums, social media, and wearables, which also enable tracking of health data [6,15]. Due to their accessibility and the potential for low-cost implementation at scale, DH tools have the capacity to easily disseminate information and interventions for behavioral change to a large audience [15]. For maternal and birth outcomes, DH tools have been applied in numerous ways to improve and manage several conditions beyond obesity. These included fertility education apps [10], tracking of symptoms and complications through a continuum from before to after pregnancy [5,7], improving the delivery of pregnancy and antenatal care [6,11,14], early labor support apps [13], and promoting postpartum health screening via DH platforms [12]. These have been widely implemented in many geographies, from European to African countries [7,10,16-18]. Accelerated by the recent COVID lockdowns [19], DH tools present an opportunity to satisfy the health care, informational, and support needs of women throughout their journey from preconception to postbirth.

To realize the potential of DH tools, attention needs to be given to their adoption and retention by the users. User engagement with the DH tool is vital in maximizing the potential benefits derived from it [18,20]. The degree of user engagement determines the depth, breadth, and length of tool use [9,21]. Studies investigating the engagement of DH tools among pregnant women report a lack of user engagement. One study examining the uptake of digital antenatal care services by pregnant mothers found poor adoption of DH due to a lack of awareness and cost barriers [16]. Other studies found that despite incentives to motivate engagement, women struggled to sustain long-term use [18]. These findings have been observed across diverse settings, ranging from high-income countries like Germany to low-income countries like Uganda. Evidently, user engagement is affected by differences in women’s accessibility to and knowledge of the DH tools [17], emphasizing the need to actively identify reasons driving unequal adoption of DH tools [22]. The needs of women evolve as they progress through their reproductive stages and thus require varying interventions at different time points [23,24].This calls for more research to understand the factors that affect the adoption and sustained use of DH tools by women to supplement current research on the perspectives of clinicians [25]. Insights gained from women through their experiences can serve to better inform DH innovators, from the early design and development process to the implementation phase.

As such, this study seeks to explore and understand the factors that influence women in their adoption and sustained use of DH tools before, during, and after pregnancy. This study provides a continuation of research from a previous study by Lee et al [26], using the same pool of data obtained from performing 44 interviews with women across preconception to postbirth phases but focusing on responses specific to technology adoption. While Lee et al [26] primarily focused on key elements in DH interventions that promote healthy behavior change in women from before to after pregnancy, this study identified 5 main themes that support DH uptake and sustained use. The translational insights derived from this study aim to inform the future design and implementation of DH tools for women across preconception to postbirth, to create a positive impact on behavioral and health outcomes in both mother and child.

## Methods

### Ethical Considerations

The study procedures were approved by the National Healthcare Group Domain Specific Review Board (reference 2021/00034). Informed consent was obtained from participants by providing the consent form during initial contact and prior to the interview session. Additionally, the researcher explained the consent form fully before participants consented. Participation was voluntary and participants could withdraw at any point of the study. There were no direct risks involved for participants and any data collected were deidentified, encrypted, and stored in accordance with the institution’s data management policies. Participants were reimbursed SG $30 (US $23.06) for their time spent on the study.

### Recruitment

This study recruited women who were trying to conceive, were pregnant at the time of contact, or had a child aged 0 to 2 years through purposive sampling from the National University Hospital and the community in Singapore (ClinicalTrials.gov Identifier: NCT05099900). Study advertisements were distributed around National University Hospital, public places (eg, bus stops, housing estates, and learning institutions), and social media platforms (eg, Telegram and Facebook). The research team provided an overview of the study to potential participants who responded to the advertisement via email, phone call, or messaging. The team screened interested participants based on the following inclusion criteria: (1) English fluency; (2) aged 21 to 45 years; and (3) actively trying to conceive or currently in first to third trimester of pregnancy or have a child aged 0 to 2 years. Participants were not eligible for the study if they met the following exclusion criteria: (1) evidence or diagnosis of cognitive impairment; (2) current diagnosis of psychiatric disorder; (3) significant hearing impairment; (4) women requiring or who had any form of assisted conception; and (5) inability to complete the study at the judgment of the clinician investigators. The research team established communication with eligible participants to schedule the interview sessions. Recruitment took place over a period of 9 months (from November 2021 to July 2022) and ended when data saturation was achieved for each group (ie, preconception, during pregnancy, and postbirth). No participants declined to participate or dropped out after consenting to the study.

### Data Collection

This study adopted a qualitative approach to explore women’s experiences from preconception to postbirth, including their DH experiences and expectations. Prior to the interview session, participants completed a web-based questionnaire (Multimedia Appendix 1) regarding their demographics and technology use patterns. Before commencing the interview, participants were informed about study goals and researchers’ interest in the research topic. All participants provided informed consent to the audio recording of the interview for the research team’s transcription purposes. A 60- to 90-minute semistructured interview was conducted either in-person or virtually via Zoom, depending on participants’ preference. Open-ended questions were used to facilitate discussion surrounding participants’ experiences with their preconception, pregnancy, or postbirth journey, and their use or expectations of DH to support their journey. Both the questionnaires and interview guide have been detailed in Lee et al [26]. Guiding topics for the interview are presented in Multimedia Appendix 1 [26]. This manuscript is focused on the DH segment of the interview, specifically exploring women’s experiences with DH and current expectations of how DH could support their maternal journey. Given that this manuscript presented a different focus on technology adoption from Lee et al [26], responses included in this work are different and were not reused between the manuscripts. All interviews were conducted in English with at least 2 researchers present. The interviewing team comprised of VVL, SV, WYN, NYL, and QYL—all female and trained in qualitative research.

Following the interview session, participants completed another web-based questionnaire (Multimedia Appendix 1) regarding their pregnancy concerns and DH expectations. All participants were reimbursed for their time. No repeat interviews were carried out and transcripts were not returned to participants. Reflective notes were taken after the interview to consider additional guiding prompts for future interviews.

All data collected, including signed consent forms, interview recordings, and questionnaire data, were deidentified, encrypted, and stored in a secure database.

### Data Analysis

For the questionnaire, descriptive analyses were conducted using SPSS (IBM Corp). Pairwise deletion was employed to handle missing data in the questionnaire responses. For the interviews, audio recordings were transcribed verbatim. Inductive thematic analysis was conducted to identify emerging and recurring themes. First, transcripts were randomly assigned to the 5 interviewing researchers to conduct primary coding to descriptively label the data. All generated primary codes were then compared and discussed among all researchers to resolve any discrepancies. Thereafter, secondary coding, where labeled data were grouped into categories, was conducted independently for each group (preconception, pregnancy, and postbirth) using Microsoft Excel. As the categories that emerged from secondary coding were similar across all 3 groups, the categories were analyzed into broader, overarching themes. The final set of codes and broader themes was concluded for the study data after discussions and iterations by all researchers. No feedback was provided by participants on the findings.

## Results

### Participant Characteristics

The cohort used in this study was previously described in Lee et al [26], where detailed demographic characteristics were provided (Multimedia Appendix 1). A total of 44 participants (age range 21-40 years; mean age 31.6, SD 4.0 years) completed the study. Participants across preconception (13/44, 29.5%), pregnancy (16/44, 36.4%), and postbirth (15/44, 34.1%) phases were recruited. Participants were Chinese (33/44, 75%), Malay (4/44, 9.1%), Indian (4/44, 9.1%), and other (3/44, 6.8%) ethnicities. In terms of education, there was an equal distribution of participants who had 15 years of education or less (22/44, 50%) and those who had more than 15 years of education (22/44, 50%). Socioeconomic status (SES) varied, with participants categorized as low SES (9/44, 20.5%), middle SES (13/44, 29.5%), and high SES (22/44, 50%). Family size ranged widely, with participants having had no children (17/44, 38.6%), 1 child (16/44, 36.4%), and 2 or more children (11/44, 25%) at the point of the study. All participants spoke fluent English.

The technology use patterns and expectations obtained from the questionnaire responses are shown in Table 1. One participant from the postbirth group was entirely excluded from the table due to nonresponse to the questionnaire. Two participants (one from the pregnancy and one from the postbirth group) had missing data due to incomplete questionnaire responses. Women indicated preferred online sources of information and information topics. For instance, Google and child development–related information were commonly perceived as a useful information source and topic respectively. Most women could accept using DH tools that required weekly logging of information, and receiving feedback based on data logged was also rated by women as a feature that they were likely to use DH tools for. Participants across the 3 phases also used a variety of DH apps, including apps for physical health, mental health, fertility, pregnancy, and child-related care, which has been detailed in Lee et al [26]. While the tables presented in Lee et al [26] focused on categories of apps being used, Table 1 shows specific technology use patterns and aspects of DH tools participants would like to see. Multimedia Appendix 1 provides a breakdown of the categories of mobile phone application use across the 3 phases.

**Table 1:** Technology use patterns and expectations of study participants from questionnaire responses.

Technology use and category			Preconception (n=13)		Pregnancy (n=16)		Postbirth (n=15)
**Useful online sources of information (n=42), n (%)**							
	Chat group	6 (46.2)		8 (53.3)		6 (42.9)	
	Online forum	11 (84.6)		7 (46.7)		8 (57.1)	
	Google	9 (69.2)		12 (80)		12 (85.7)	
	Mobile phone apps	5 (38.5)		10 (66.7)		9 (64.3)	
	Social media	8 (61.5)		9 (60)		7 (50)	
**Acceptable frequency of logging information (n=42), n (%)**							
	Daily	5 (38.5)		5 (31.3)		4 (30.8)	
	Weekly	8 (61.5)		10 (62.5)		7 (53.8)	
	Monthly	0 (0)		1 (6.3)		1 (7.7)	
	Biweekly	0 (0)		0 (0)		1 (7.7)	
**Likelihood of using DH^a^ platform for respective features (n=43; Scoring: 0=Extremely unlikely, 2=Somewhat unlikely, 3=Neither unlikely or likely, 4=Somewhat likely, 5=Extremely likely), mean (SD)**							
	Complete questionnaires	2.85 (1.07)		2.38 (0.96)		2.53 (1.03)	
	Log physical health data	3.46 (0.52)		3.00 (0.52)		2.93 (1.14)	
	Log mental health data	2.69 (0.95)		2.75 (0.78)		2.36 (1.15)	
	Feedback based on data logged	3.00 (0.58)		3.13 (0.50)		3.07 (0.83)	
	Lifestyle guidelines and advice	3.38 (0.65)		3.00 (0.37)		3.14 (0.86)	
	Peer support	2.85 (1.07)		2.88 (0.89)		3.21 (0.80)	
	Breastfeeding and weaning information	3.08 (1.12)		3.00 (0,73)		3.5 (0.65)	
	Connect to wearables	2.54 (1.27)		2.75 (1.29)		3.14 (1.10)	
	Pair with digital tools	2.54 (1.33)		2.44 (1.21)		2.71 (1.33)	
**Preferred information topics on DH platform (n=42), n (%)**							
	Developmental information of child	13 (100)		16 (100)		11 (84.6)	
	Mental health resources	6 (46.2)		11 (68.8)		11 (84.6)	
	Physical activity	10 (76.9)		13 (81.3)		8 (61.5)	
	Helpline and health provider contact details	10 (76.9)		9 (56.3)		10 (76.9)	
	Others	Types and cost of local fertility or maternity services		Postpartum recovery resources, gestational diabetes mellitus meal plan suggestions, activities with newborn, coping with changes after delivery (ie, physical, mental, work, and social aspects)		—^b^	

^a^DH: digital health.

^b^Not applicable.

### Interview Data

**Figure 1:**
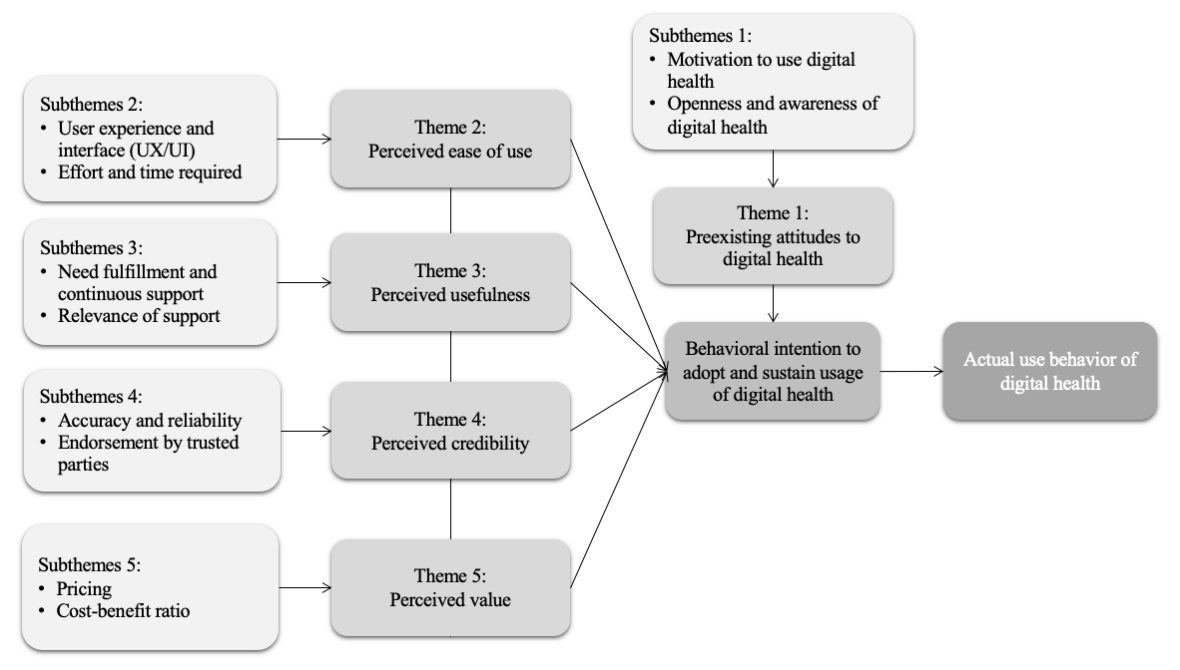
Factors influencing women’s behavioral intention to adopt and sustain use of digital health tools.

A total of 5 themes and 10 subthemes were identified as factors that motivate or hinder women’s behavioral intention toward adoption and sustained use of DH tools (Figure 1).

#### Theme 1: Preexisting Attitudes Toward DH

Existing attitudes are defined as women’s motivation to use DH tools, as well as their openness and awareness of DH tools.

##### Motivation to Use DH

Prior experiences and understanding of DH shaped women’s current motivations to adopt DH tools. Women expressed having low motivation due to previous unpleasant experiences with DH tracking tools whereby tracking lifestyle metrics such as diet, physical activity, and water intake felt like a chore. Some women disengaged because they lacked the motivation to enter data manually, while others found it stressful to adhere to the requirement of regular data entry.

Last time I used to track my water intake, but got a bit lazy...because [you’ve] got to key in manually so I stopped that.

Participant 5, preconception

Previously I was tracking my diet but it got too troublesome.

Participant 18, postbirth

After like one, two months, I start to get very stressed in order to meet this routine [and] having to comply to the app, I think that would be more stressful to me...Do a food diary, nope that’s [going to] be more stressful...

Participant 41, preconception

Such poor motivation was also attributed to poor understanding of the impact of lifestyle factors on maternal and child health, and how tracking lifestyle metrics might be useful.

I don’t really know how sleep can impact, [so] I wouldn’t really track sleep.

Participant 33, preconception

So like of course [diet tracking is] good to have but I don’t have [the] confidence I will use it. I also don’t know what [features of diet tracking are] good.

Participant 18, postbirth

##### Openness and Awareness of DH

Women were generally open to using DH tools to manage their pregnancy journey if they were aware of such tools, provided that the tool met their need for it to be easy to use, require little time, and provide health monitoring benefits.

But of course, if there’s an app that can help me monitor my health better, I might be willing to try it.

Participant 34, preconception

I think if it was too complex to use, or if the app made me spend too much time on it, then I might not want to be so hooked on my digital device, especially if I have young kids.

Participant 23, postbirth

Women also suggested that outreach efforts to increase knowledge and awareness of a DH tool and its benefits might also encourage the adoption of the tool.

I think that if they come and engage me...like do you know that this app allows you to do this and do that. I’m like “okay!”

Participant 12, preconception

#### Theme 2: Perceived Ease of Use

Based on the interviews, DH tools’ perceived ease of use was contingent upon factors such as user experience and interface of the digital platform, as well as how much effort and time were required from the user in their use of the DH tool.

##### User Experience and Interface (UX/UI)

Women’s intention to continue the use of a tool after initial adoption was highly dependent on the user experience and interface of the DH tool. Unappealing interfaces that were challenging to navigate, slow loading times, or discomfort in user experiences were major sources of frustration that led to disengagement with a digital tool.

Just [that] the outlook was not very appealing...maybe the colour, the outlook, the way it is placed, not very user friendly. It’s a bit confusing on how to use it.

Participant 31, during pregnancy

For me like the UX is important also, the ease of use and the features and functionalities of the app.

Participant 4, during pregnancy

When I needed to track those information [for my baby], I downloaded a few apps to try.

If its’ hard to use, or the interface is not so...erm I don’t feel comfortable, I’ll just straightaway delete. Until I find one that’s comfortable, then I just stick to it.

Participant 1, postbirth

Hardware and software issues of a DH tool such as the extent of battery consumption, data use, storage space, and data syncing problems lowered the likelihood of adoption and sustained use. Women were also highly likely to delete an app if they experienced frequent technical glitches.

[I used to use] HealthHub, but I’m not using [it] now because the syncing has some issues.

Participant 37, preconception

Too much data, memory space, [and also] it will be good if [it] doesn’t drain too much battery. I think Fitbit drains quite a bit [of battery life].

Participant 5, preconception

What would stop me [is] if it’s always buggy and always hanging, and then like half the time you can’t get the app to work, then obviously I would just delete it.

Participant 38, postbirth

##### Level of Effort and Time Required

The perceived ease of use was influenced by the level of effort and time required on the part of the user. Women identified several barriers that prevented continued use of a DH tool, including data entry that required too much time and effort, especially postbirth when women were busy caring for their children. DH tools with a steep learning curve and long onboarding time in the initial stage of use were also the reasons that led women to disengage from digital tools.

But after a while I stopped [tracking my diet] because I feel that it was a bit troublesome to have to key in what I eat, then find the exact food and portion size that I ate. I mean, although that did help me to manage my diet, but it was troublesome for me to use in the long run.

Participant 18, postbirth

So in the beginning I use [an app for breathing exercises] more, but I think when [my child] came, well I had no time to do that anymore

Participant 8, postbirth

When you first [begin to use] an app, [you] probably need to load quite a bit of information in there. So if that first stage [takes] me a little longer to actually set it all up, I might just give up in the end.

Participant 34, preconception

#### Theme 3: Perceived Usefulness

Another crucial determinant in women’s adoption and ongoing use of DH tools was perceived usefulness, which was shaped by the extent of need fulfillment and continuous support provided, as well as the relevance of support.

##### Need Fulfillment and Continuous Support

It was important to women that DH tools fulfilled their unmet needs and delivered comprehensive support. The idea of providing an all-in-one solution was especially appealing, due to issues like app fatigue as women found it tedious to manage several apps for different health-related functions, or having to filter through overwhelming amounts of information on the internet. Having an all-in-one support tool was pointed to as an enabler of better access to quality information and increased ease in tracking health and lifestyle metrics.

I would say the only thing that would make [the DH tool] superior would be the fact that it has everything I need. So I can just delete my Flo app, I don’t even have to do my Google search anymore. I mean [I can] reduce my Google search at least.

Participant 35, preconception

Yea, as in I think that would be helpful because sometimes along the way in the journey then you have questions, and you don’t pre-empt the questions way before, so if the, the app covers the whole span then you can obviously uh, look through it at the different stages that you’re at in your journey

Participant 26, postbirth

Furthermore, it was critical that DH tools delivered complementary support to the current continuum of care provided by the health care system. This was especially prominent for women in the pregnancy and postbirth phases, as many of their needs included touch points with the health care system, such as understanding their pregnancy trimesters or their child’s development through doctor consultations or health booklets. Offering complementary support emerged as a feature to increase the likelihood of DH adoption. For example, scheduling appointments and integrating DH records of the child were mentioned as useful tools.

If I can use this app as like an all-in-one [tool], [including] scheduling appointments [or] even uploading my ultrasound photos...And you can download the digital copy of [the ultrasound scan], instead of having just the physical [copy], which you might lose or it might degrade over time. These ultrasound scans are quite precious to like the family during the pregnancy [because] that is [the] only time where you can like see the baby.

Participant 4, during pregnancy

##### Relevance of Support

Women highlighted the importance of the relevance of support provided by DH tools. They valued being able to receive tailored information based on their individual needs and preferences. This included information that was relevant to women’s current phase in the pregnancy journey, topics of interest, or areas of child development in which they had concerns at that time.

What would stop me from using the app is if I’m not interested to know about that particular topic or maybe I don’t see that my child has any issues at all in that aspect, then I wouldn’t bother to check.

Participant 26, postbirth

The desired feedback from tracking maternal indicators was envisioned as being aware of women’s conditions and sensitive to their emotional well-being, ensuring that it would not induce additional stress.

It really depends on the circumstances. Let’s say I’m producing a lot of breast milk [and] I’m keying it into the app, then I wouldn’t mind seeing it. But let’s say I’m a mother who doesn’t produce enough [breastmilk] and I’m keying it into the app. Then, I look at it and I start to feel depressed. Then, I probably would not enter it again.

Participant 33, preconception

The relevance of support was particularly important as women were often overwhelmed by the sheer amount of information available through technology. Often, such “knowledge burden” (participant 30) became unhelpful and a source of anxiety, as women needed to have sufficient mental capacity to filter through information and determine what was useful and appropriate for their circumstances.

Maybe you read too much and then you give yourself anxiety. Let’s say [the information states] you cannot eat [too much of this], then you’ll [wonder if you’re] eating too much. So sometimes, in a way it’s more like [a] knowledge burden. Yeah but you need to know how to filter.

Participant 30, preconception

#### Theme 4: Perceived Credibility

The extent to which DH tools were deemed credible was described to have a notable impact on their adoption and long-term use. Accuracy and reliability of information and support, along with endorsement by trusted parties through social networks, played a role in shaping the perceived credibility of a DH tool.

##### Accuracy and Reliability

Women indicated the need to perceive the information and support provided as accurate and reliable. Women brought up challenges in navigating conflicting information from online sources, their social networks, and health care professionals. They suggested that it was essential for DH tools to provide accurate information that aligned with clinical recommendations from health care professionals. Perceived reliability of information was also influenced by the source of information, as women expressed greater trust in the advice provided by medical professionals.

Sometimes [information] can be contradicting so I’m not sure which source to trust.

Participant 9, during pregnancy

Let’s just say they have a moderator, a medical professional [in the online forum], that would answer questions here and there, that could be a game changer. Because then, I would look out for this badge – okay this [medical professional] says this. So, that would be an edge over a sea of apps.

Participant 14, during pregnancy

But I was hoping [for information about] medication [that you take] before pregnancy or after pregnancy, or medication [you take] while you are pregnant, breastfeeding and stuff. But sometimes, [information] is inaccurate. Let’s say there’s some medication, [like] Panadol [that] you can’t take while breastfeeding, but the doctor [says] you can take while you are breastfeeding...Trust the doctor or trust the app?

Participant 42, postbirth

##### Endorsement by Trusted Parties

Women were more inclined to use DH tools recommended by trusted networks. Most women agreed that gaining awareness about DH tools from their clinicians or health care providers was important in influencing their decision to adopt them.

Definitely if a healthcare provider recommended it. So a lot of the things I have on my phone are to do with [physiotherapy] and all that, they’ve all come from the [physiotherapist], the lactation consultant, birth class teacher.

Participant 7, during pregnancy

Social networks that women were already situated in also had a strong influence on their decision-making. Recommendations via word-of-mouth from friends, female communities on online support groups or forums, and key influencers on social media platforms were persuasive in encouraging the adoption of DH tools. Additionally, it was important that these trusted parties were situated in the local context. Another commonly brought-up factor that influenced women’s choice of DH tools was the sharing of experiences, such as how other women or mothers overcame their struggles with the support of DH tools. Such positive reviews were perceived as credible information that affirmed DH tools’ ability to provide adequate and effective support to help women manage similar challenges.

Asian Parent [online platform with parenting-related content] was recommended because I know of friends who used it. So, I think word of mouth is always more powerful.

Participant 14, during pregnancy

Recommendations from friends or mommies. If I was on the internet, and it popped up, [saying that]this is the number 1[app]. Not trending app but in your area, maybe, within Asia or within Singapore, this is the app most mommies are using.

Participant 7, during pregnancy

Women also considered satisfaction ratings and reviews by other users on the internet or application download platforms when deciding which DH tools to adopt.

If it’s on Play store, they have reviews and stars, so I kind of read those first. I mean some of them have issues like after an update, it just [does not] work anymore, then I just don’t download [those apps]. But I generally go for those that are generally highly ranked. If they are highly ranked, but their latest reviews are actually quite bad, then I don’t really [download those too]. Then if I do have one in mind already, I kind of go and Google for that specifically to see what are the reviews.

Participant 34, preconception

#### Theme 5: Perceived Value

The pricing of a DH tool, together with the balance of costs and benefits, played a pivotal role in shaping the perceived value of the tool.

##### Pricing

Women raised concerns regarding DH tools that required payment as there was a plethora of free applications and information on the internet. Specifically, DH tools that required an upfront fee were a strong deterrence to adoption as women preferred to experience the tool to understand its utility before deciding on the financial commitment.

[I think] what would stop [me from using an app would be] the pricing. So, I’m not too sure how much I would be willing to pay for such an app, because like [I said], everything can be found on the Internet.

Participant 5, preconception

I think if it’s paid, like straight-up a paid app, and I am not sure what the features are, that might prohibit me from paying for it or downloading it.

Participant 4, during pregnancy

Instead, women were more open to using DH tools that were free but had paid features embedded within the tool, as they were able to make more informed purchase decisions based on its value.

But if it’s like Flo, [which is] free to download and then there is paid features that I feel like might be helpful, then I might consider paying for it. So, price might be a challenge.

Participant 4, during pregnancy

##### Cost-Benefit Ratio

Women emphasized that their willingness to bear the expenses of DH tools is dependent on whether the cost is supported by its perceived benefits. One feature that women were willing to pay a premium for included teleconsultation features with health care professionals and receiving medical advice.

If it’s just to calculate my ovulation, I don’t think [I will pay for an app]. But if it’s an app that has [in-built] chatbots, where I am able to consult doctors or fertility experts on any subject, then yes, I will [pay for the app].

Participant 39, preconception

Other factors, such as perceived usefulness and perceived credibility would increase the perceived value of DH tools. Benefits such as continuous support from preconception to postbirth, including long-term support for their child’s health and development, and personalization were brought up to outweigh price considerations. The ability of the DH tool to provide an all-in-one comprehensive platform—integrating health records and incorporating functions of other health apps to reduce app fatigue—was pointed out to further enhance the tool’s value.

[I would pay for the app] if it’s going to override [other apps] like Health Hub. Otherwise, it would kind of defeat the purpose if I’m on this app to track my baby’s progression, [but] at the same time I have to track my baby’s health records on Health Hub.

Participant 41, preconception

In addition, the cost of DH tools needed to be justified by its credibility, through evidence-based information and support.

It depends on the [expanse] of the app. So if it follows me until the baby is two years old, then the amount that I would pay would be [different from] if it allows me to follow through to 8 years instead.

Participant, 41, preconception

To garner a premium subscription rate, the app must really be personalized, or must really have sufficient evidence, [so] that it’s worth the subscription fee.

Participant 5, preconception

## Discussion

### Principal Findings

Our study identified the major themes that influenced women’s adoption of DH tools from before, during, to after pregnancy. Depending on the stages they were in, women applied different considerations of those themes. For instance, the level of time and effort required to engage in DH tools was more salient to women as they were unlikely to devote time out of their busy schedules preconception, or out of their priorities to caring for their newborn postbirth. By accounting for the varying challenges faced by women in different stages, DH tool developers can improve user engagement with the tool and sustain use over time.

### Unified Theory of Acceptance and Use of Technology (UTAUT) 2 Model

The 5 main themes and 10 subthemes discussed were mapped based on existing literature on IT use, specifically the Unified Theory of Acceptance and Use of Technology 2 (UTAUT 2) model. UTAUT 2 consists of 7 factors identified to contribute to the acceptance of technological tools: performance expectancy, effort expectancy, social influence, facilitating conditions, hedonic motivation, price value, and habit [27]. UTAUT 2 has its roots in the UTAUT model, which was formed by empirically comparing 8 other models and deriving the primary factors affecting technology use [27]. UTAUT 2 expands UTAUT with factors focused on behavioral intention to use the technology [27].

The themes of perceived usefulness and perceived ease of use identified in our study can be mapped to performance expectancy and effort expectancy respectively in UTAUT 2. The quality of information women received and the extent to which they believed the information fulfilled their needs determined their perceived usefulness of the DH tool and was therefore likely to influence their intention to adopt it. As women progress through different stages, their informational needs would change accordingly (eg, different information relevant to specific trimesters across pregnancy) [23,24]. Having a DH tool that caters to information and features specific to a particular stage in time would naturally improve engagement [26]. The predictability of informational needs from before to after pregnancy can offer a guide to DH developers in providing relevant content to women, thus improving their perceived usefulness of the DH tool. Additionally, enhanced antenatal education for women on improving long-term health outcomes would serve as motivation for higher quality information [16], thus highlighting the importance of perceived usefulness in the use of DH tools. Likewise, for effort expectancy, their experiences and the resources required of them in terms of time and effort would determine their perceived ease of use of the tool.

Under the theme of perceived credibility, there were two subthemes that emerged as follows: (1) the endorsement by trusted parties and (2) the accuracy and reliability of information. The subtheme of endorsement by trusted parties can be likened to the factor of social influence under the UTAUT 2 model. In addition to social influence being a key factor for the adoption of DH tools (as in UTAUT 2), the trust it invokes and the way it is marketed also influenced women’s intentions to adopt it. Interestingly, while the UTAUT 2 model predicts the significance of the social influence factor when tool use is mandated [27], in our study, endorsement by trusted parties was highlighted where the use of DH tools by women was not mandatory. The subtheme on the perceived quality of information is not directly linked to any of the factors in UTAUT 2. It is plausible that women in our study who looked for information online were exposed to information that was not verified or reliably accurate [28]. This contrasted with other available trusted sources and may have led to the emergence of this specific subtheme. Overall, while perceived credibility is not a factor in UTAUT 2 in its entirety, current literature shows the rising importance of perceived credibility as a predictor of intention, over factors such as DH literacy in DH tool adoption [22,28]. The theme of perceived credibility and its relationship with social influence prompts further research into the significance of social influence and its nuances for technology adoption specific to a particular deployment context. In one example, public health care is a widely provided good in Singapore, and Singapore residents express trust in public health care and government services. As such, based on our study, promotion, and endorsement by government or public health care professionals may support the perceived credibility of the DH tool and in turn, promote adoption of the tool [29].

Another theme that emerged was perceived value, similar to the factor of price value on the UTAUT 2. Perceived value focuses on the subjective value that women assign to the DH tool, rather than the absolute price value of it as defined in UTAUT 2. Women revealed that they often performed a subjective cost-benefit analysis before deriving the absolute price value they were willing to pay for the DH tool. In addition to considering the pricing of the tool, women would weigh costs against the subjective benefits they derive from the tool. When pricing the tool, it is important to understand what women take into consideration to estimate their willingness to pay in any given tool deployment context. In Singapore, as public health care programs are subsidized or provided for free, women also tend to expect the prices of tools to be subsidized, or free-of-charge [30,31].

Another theme that emerged in our study, women’s preexisting attitudes toward DH, can be reflected by the UTAUT 2 factor of facilitating conditions, stipulating that women’s attitudes and their prior experiences would influence their perception of the new tool. Women in the preconception, pregnancy, and postbirth groups collectively used over 34 different DH apps for a variety of functions such as physical health monitoring; government and public health–related; mental health; and fertility-, pregnancy-, and child-related health (Multimedia Appendix 1). This indicates that women are experienced with DH use, which is aligned with the growing prevalence of DH use by women in Singapore [32]. Women who have had negative experiences with app use may hesitate when faced with another similar app again [33]. Analogically, positive experiences may reinforce positive attitudes toward a new DH tool.

Out of the 5 themes from our study, 2 themes can be fully (ie, perceived ease of use and perceived usefulness) and 3 themes can be partially (ie, perceived credibility, perceived value, and preexisting attitudes to DH) mapped to the factors listed in UTAUT 2 [27]. In addition, the model has to be contextualized to the deployment environment, and the differences between the themes that emerged in our study and the factors of UTAUT 2 warrant further research. Additionally, there were 2 other factors in the UTAUT 2 model that did not emerge as themes in our study: hedonic motivation and habit. It is possible that while habitual use may determine DH use, women could end up switching between DH tools catering to different needs too often to form a habit. Women may also perceive the use of DH tools to be limited to their reproductive journey and hence may not form a habit as opposed to DH tools for long-term health [34]. It is also possible that while those factors were at play, the participants’ lack of awareness or expressiveness did not allow us to capture these factors with the deployed methodology. This area warrants further research. Nevertheless, UTAUT 2 shows high potential as a model used to understand women’s DH engagement during their reproductive journey.

### Adoption Intention and Sustained Use

The behavioral intention to use a DH tool has been previously shown to vary across the user journey, from the initial adoption to continued use [35]. It was the case for women in our study as well. The 5 identified themes exert different influences on their decision-making at different timepoints in their journey. Themes such as preexisting attitudes toward DH*,* perceived credibility, and perceived value are more pertinent in influencing adoption intention. For instance, in the early adoption stages, women’s motivation to adopt a DH tool was more significantly influenced by their preexisting attitudes toward DH, shaped by previous unpleasant experiences or lack of understanding of DH tools. Women also sought recommendations from their surroundings to form the perceived credibility of the DH tool in determining if they would want to adopt it. Similarly, the UTAUT 2 model on technology adoption identified social influence to be pertinent to women, especially in the early stages of adoption [27]. This was confirmed by our study where women mentioned that recommendations about DH tools from their trusted social networks would have enhanced their early adoption of the DH tool. Additionally, the theme of perceived value has been shown to factor into user intention [36], where women preferred free-to-use apps with only a small minority willing to pay for the tool.

Themes such as perceived usefulness and perceived ease of use are relevant to both early adoption and sustained use of the DH tools. In terms of early adoption, the perceived usefulness and perceived ease of use have been shown to influence the intention to use [35]. The ability of the tool to meet women’s needs in specific phases and transition across phases (eg, from pregnancy to postbirth), and a smooth experience using the tool emerged as desired factors that motivated sustained use in the long run. Additionally, women in our study highlighted the expected effort required for the DH tool as a determinant of its long-term use. This could be due to the changing needs and bandwidths of women over different phases of motherhood, which affect their capacity to manage the expected effort in sustained use of a DH tool, which calls for individualized requirements and informational needs [23,24]. As women navigate each phase, this would necessitate adapting to new features of the tools and resources required of them. The relatively rapid and predictable timing of the transition from one phase to the next is inherently useful and can be harnessed to sustain engagement from one phase to the next. Interestingly, prior studies suggest that effort expectancy is not a predictor of continuous use [35], and such discrepancy would require further research.

There are many studies examining the difference between the early adoption and sustained use of information systems [35,37]. There is a gap, however, in the understanding of these 2 elements for DH tools adoption by women from before to after pregnancy. In this study, we propose how the 5 discovered themes interact with these 2 elements and note the role of the deployment context in the realization of these elements.

### Future Directions

This study works as a foundational inquiry into understanding women’s needs and expectations, in line with the principles of co-design [38]. The potential next step could include the generation of the content-strategy-design guide—a deep dive into the specific components of the tools, such as content, appearance, workflow, and integration with the system and the existing user journey [39]. By eliciting themes that are the most salient to women in their reproductive journey, these insights can serve to inform DH tool developers in designing ways to improve engagement with a DH tool. Such a strategy has been used before, where qualitative methodology was employed to co-design a DH tool for women in pregnancy [30]. Table 2 summarizes the insights and recommendations that emerged from our study. Along with technology expectations in Table 1, this could provide specific guidance in designing frequency of use, sources of information, and the preferred information topics at each phase from preconception to postbirth.

**Table 2:** List of recommendations based on women’s identified challenges and motivators in the adoption and sustained use of digital health tools.

Theme and subtheme			Recommendation
**Perceived ease of use**			
	User experience and interface (UX/UI)	Consistent updating and maintenance of apps	
	Level of effort and time required	Adjusting the use of the app to user’s availability	
**Perceived usefulness**			
	Need fulfillment and continuous support	All in one tool allowing women to use it consistently from preconception to postbirth, covering multiple functions	
**Perceived credibility**			
	Endorsement by trusted parties	Encouraging adoption by getting trusted endorsements from fellow mothers and health care providers (gynae, etc)	
**Perceived value**			
	Pricing	Accounting for localized context where users expect free-to-use apps	

### Limitations

As 79.5% (35/44) of our participants were of middle to high SES, the results of our study may have limited generalizability, especially stemming from their prior familiarity with technology. Additionally, the recruitment criteria included English fluency. Further research can investigate the perceptions of lower SES groups and non-English speakers to examine the validity of the identified factors affecting sustained adoption. Last, women who had used or were using assisted conception were excluded, similarly to women who had current diagnoses of psychiatric disorders. These women might face vastly different struggles and considerations to the population we examined which could potentially be supported by various DH tools as well. This calls for future research to examine the applicability of our findings to these populations of women from before to after pregnancy.

### Conclusion

The study examined the factors affecting sustained adoption of DH tools for women from before to after pregnancy and mapped them according to the UTAUT 2 model. Our study shows that the UTAUT 2 model can broadly reflect the adoption of DH tools in women from before to after pregnancy. Specific considerations of the cultural implications are needed in its application to the context of Singapore, a highly tech-pervasive Asian society, which is different from the European and African contexts where prior DH adoption studies have been conducted. Additionally, understanding the interactions of the themes with early adoption and sustained use could support DH design in ensuring both uptake and long-term use. There are various considerations to be noted in the design of DH tools for women’s preconception to postbirth to promote engagement, and tool design should be a regular iterative process to continuously improve the ability of the tool to meet women’s needs.

## Appendix

Multimedia Appendix 1 Annexes of pre- and postinterview questionnaires, interview framework, demographic data, and mobile application usage data of participants.

## Abbreviations

DH: digital health
SES: socioeconomic status
UTAUT: Unified Theory of Acceptance and Use of Technology

## Data Availability

The data sets used or analyzed during this study are available from the corresponding author on reasonable request.
