# Supplementary material for: Shaping Adoption and Sustained Use Across the Maternal Journey: Qualitative Study on Perceived Usability and Credibility in Digital Health Tools
Source: JMIR Hum Factors. 2024 Oct 1;11:e59269. doi: 10.2196/59269 (PMC11480679; doi:10.2196/59269)
Supplement: Multimedia Appendix 1 [file humanfactors_v11i1e59269_app1.docx]

**Annex A**

**Pre- and Post-interview Questionnaires [22]**

**Pre-interview Questionnaire**

Employment status and housing type

1. What is your current status of employment?
   - Employed full-time
   - Employed part-time
   - Unemployed
   - Others – please state: _____
2. What is your current occupation? Please state: __________
3. What type of housing are you currently living in?

- 1- tor 2-room public housing
- 3-, 4- or 5-room public housing
- Private housing (e.g., condominiums, landed properties, etc.)

Daily schedule and hobbies outside of work

1. On average, how much time do you spend away from work in a day (e.g., on hobbies, relaxation, etc.)?
   - Less than 2 hours
   - Between 2 to 5 hours
   - More than 5 hours
   - N/A
2. How often do you engage in physical activities (e.g., exercise) in a week?

- 0 to 2 times a week
- 3 or 4 times a week
- 5 or more times a week

Support and assistance

1. Please rank your sources of support during this time, e.g., pregnancy, childcare (1 being the highest).
   - Partner
   - Parent(s)
   - Sibling(s)
   - Extended family
   - Friends
   - Community
   - Healthcare system
2. Please rank the following types of assistance you find useful to have during this time (1 being the highest).
   - Advice on mental health
   - Advice on physical health
   - Advice on lifestyle (e.g., diet)
   - Parenting tips
   - Medical-related advice for mothers/mothers-to-be
   - Medical-related advice for child

Thank you for the participation thus far! The last part of the survey looks at your usage of technology in everyday life.

Use of technology

1. Do you use the Internet to search for pregnancy/maternal-related information or advice?
   - Yes
   - No
2. Which of the following online sources do you find useful?
   - Chat groups (e.g., Telegram, WhatsApp chat groups)
   - Online forums
   - Google search (e.g., Wikipedia)
   - Mobile phone apps
   - Social media platforms (e.g., Instagram, Facebook, YouTube)
   - None, I don’t find online resources useful.
   - Others – please state: __________
3. Please rank the following in the order you would seek for pregnancy/maternal-related information or advice (1 being the highest).
   - Internet (e.g., Google, websites, media articles, online forums)
   - Mobile phone apps
   - Social media platforms (e.g. Instagram, Facebook, YouTube)
   - Healthcare professionals (e.g., doctors, nurses, etc.)
   - Alternative health practitioners (e.g., TCM, Ayurveda)
   - Family
   - Friends
4. What are the health apps (e.g., fitness, lifestyle, pregnancy, childcare-related apps) you use generally? Please list: __________

**Post-interview Questionnaire**

1. What are some of the pregnancy-related concerns that have crossed your mind before? (Select all applicable)
   - Fear of gaining weight
   - Pre-/post-partum depression
   - Anxiety from trying to get pregnant or the thought of childcare
   - Stretch marks
   - Others – please state: __________________
2. How likely would you use a digital health platform that

Requires completing standard questionnaires (e.g., food intake, physical activity and psychological health) at regular intervals

- - Extremely likely
  - Somewhat likely
  - Neutral
  - Somewhat unlikely
  - Extremely unlikely

Offers the function of logging in physical health data (e.g., weight, diet and physical activity) for the purpose of tracking and monitoring

- - Extremely likely
  - Somewhat likely
  - Neutral
  - Somewhat unlikely
  - Extremely unlikely

Offers the function of logging in mental health data (e.g., stress, mood) for the purpose of tracking and monitoring

- - Extremely likely
  - Somewhat likely
  - Neutral
  - Somewhat unlikely
  - Extremely unlikely

Sends feedback to you whenever you key in your physical and/or mental health information

- - Extremely likely
  - Somewhat likely
  - Neutral
  - Somewhat unlikely
  - Extremely unlikely

Offers lifestyle guidelines and advices

- - Extremely likely
  - Somewhat likely
  - Neutral
  - Somewhat unlikely
  - Extremely unlikely

Offers peer support

- - Extremely likely
  - Somewhat likely
  - Neutral
  - Somewhat unlikely
  - Extremely unlikely

Offers information on breastfeeding and weaning

- - Extremely likely
  - Somewhat likely
  - Neutral
  - Somewhat unlikely
  - Extremely unlikely

Can be connected to wearable devices to track activities (e.g., weight, steps taken, sleep)

- - Extremely likely
  - Somewhat likely
  - Neutral
  - Somewhat unlikely
  - Extremely unlikely

Can be paired with digital tools (e.g., Bluetooth-enabled weighing machine) for the ease of tracking and monitoring

- - Extremely likely
  - Somewhat likely
  - Neutral
  - Somewhat unlikely
  - Extremely unlikely

1. Which of the following frequency is acceptable to you with regards to logging in of information?
   - Daily
   - Weekly
   - Monthly
   - Others – please state: ____________________
2. What type of topics do you wish to be able to access in a digital health platform?
   - Developmental information of the foetus/baby
   - Mental health resources
   - Physical activity ideas and videos
   - Helpline and health provider contact details
   - Others – please state: ____________________
3. I prefer to stay anonymous when interacting with peers on a digital health platform
   - Strongly agree
   - Agree
   - Neutral
   - Disagree
   - Strongly disagree

**Annex B**

| Interview framework [22]. | |
| --- | --- |
| Topic | Guide for discussion |
| Women’s perspectives regarding pre-conception/pregnancy/post-birth journey | - Experiences in their current phase (challenges, positives, lifestyle changes) |
|  | - Information-seeking and support system (sources and utility of information and support) |
|  | - Touchpoints with the healthcare system (for women and/or their children) |
|  | - Current quality of care and expectations of the healthcare system |
| DH usage and expectations | - Experiences with DH (e.g., wearables, mobile phone apps) |
|  | - Opinions on current DH tools for pre-conception, pregnancy or post-birth |
|  | - Ideal features for DH tools for pre-conception, pregnancy or post-birth |
|  | - Facilitators and barriers to adoption and sustained usage of pre-conception, pregnancy or post-birth DH tools |

**Annex C**

| Demographic data of study participants from questionnaire responses and interviews. [22] | | | | |
| --- | --- | --- | --- | --- |
| Demographics |  | Pre-conception | Pregnancy | Post-birth |
| N |  | 13 | 16 | 15 |
| Age range (years) |  | 21–39 | 27–39 | 25–40 |
| Ethnicity (n) | Chinese | 11 | 11 | 11 |
|  | Indian | 0 | 1 | 3 |
|  | Malay | 2 | 1 | 1 |
|  | Others | 0 | 3 | 0 |
| Education (n) | 15 years or less | 7 | 10 | 5 |
|  | More than 15 years | 6 | 6 | 10 |
| Socioeconomic status (n) | Low | 1 | 4 | 4 |
|  | Middle | 5 | 4 | 4 |
|  | High | 7 | 8 | 7 |
| Number of children (n) | 0 | 7 | 10 | 0 |
|  | 1 | 5 | 4 | 7 |
|  | 2 or more | 1 | 2 | 8 |

**Annex D**

Mobile phone application usage patterns of study participants from questionnaire responses. [22]

| Technology usage |  | Pre-conception | Pregnancy | Post-birth |
| --- | --- | --- | --- | --- |
| Mobile phone application usage (N=42) | Physical wellbeing health apps | Apple Health, Fitbit, Samsung Health, Poop diary | AIA Vitality, Fitbit,  NBuddy | Calm: Sleep, Google Fit, Meditation, MyFitnessPal, Prenatal Yoga Down Dog, Samsung Health, Upside Motion |
|  | Government health apps | HealthBuddy, HealthHub, Healthy365, Lumihealth | Healthy365 | HealthBuddy, HealthHub, Healthy365 |
|  | Mental health apps | Headspace, Mindfulness apps | - | - |
|  | Fertility or pregnancy or child-related apps | Baby Read, Fertility Friend, MyFertility | BabyCenter, Mama & Baby, Pregnancy+, Pregnancy After Loss, The Asian Parent, What to Expect, Wonder Weeks | BabyCenter, BabySparks, Baby Tracker, Huckleberry, Little Family Room, Pregnancy, The Asian Parent, The Wonder Weeks, What to Expect |
